# Biologic and Genetics Aspects of Chagas Disease at Endemic Areas

**DOI:** 10.1155/2012/357948

**Published:** 2012-03-08

**Authors:** Marilanda Ferreira Bellini, Rosana Silistino-Souza, Marileila Varella-Garcia, Maria Tercília Vilela de Azeredo-Oliveira, Ana Elizabete Silva

**Affiliations:** ^1^Department of Especial Education, UNESP São Paulo State University, 17525-900 Campus Marília, SP, Brazil; ^2^Department of Biology, UNESP São Paulo State University, 15054-000 Campus São José do Rio Preto, SP, Brazil; ^3^Medicine/Medical Oncology, University of Colorado Health Sciences Center, Aurora, CO 80045-0511, USA; ^4^University of Colorado School of Medicine, Anschutz Medical Campus, Research Center 1 South Tower, Mail Stop 8117, Aurora, CO 80045-0511, USA

## Abstract

The etiologic agent of Chagas Disease is the *Trypanosoma cruzi*, transmitted through blood-sucking insect vectors of the Triatominae subfamily, representing one of the most serious public health concerns in Latin America. There are geographic variations in the prevalence of clinical forms and morbidity of Chagas disease, likely due to genetic variation of the *T. cruzi* and the host genetic and environmental features. Increasing evidence has supported that inflammatory cytokines and chemokines are responsible for the generation of the inflammatory infiltrate and tissue damage. Moreover, genetic polymorphisms, protein expression levels, and genomic imbalances are associated with disease progression. This paper discusses these key aspects. Large surveys were carried out in Brazil and served as baseline for definition of the control measures adopted. However, Chagas disease is still active, and aspects such as host-parasite interactions, genetic mechanisms of cellular interaction, genetic variability, and tropism need further investigations in the attempt to eradicate the disease.

## 1. Chagas Disease

### 1.1. Epidemiology and Clinical Outcomes

Chagas disease, also called American trypanosomiasis, remains an epidemiologic challenge more than one hundred years after its discovery by Carlos Chagas [1]. It is estimated that 12–14 million people are infected with *Trypanosoma cruzi *in Latin America where the disease is endemic, and 75–90 million are exposed to infection [1, 2]. Less frequently, infection occurs through blood transfusion, vertical transmission (from infected mother to child), or organ donation [3].

In 2008, it was estimated that more than 10 thousand people were killed by Chagas disease [3]. In Brazil, the infection has already afflicted about 2.5 million individuals [4] despite the success of control measures responsible of elimination of domestic and peridomestic colonies of vector and monitoring of blood banks, which reduced incidence by approximately 70% in the Southern Cone countries. Due to the intense population migration and mobility, Chagas disease has spread in North America and Europe and is now global [5, 6].

Chagas disease is characterized by a wide spectrum of clinical outcomes, ranging from absence of symptoms to severe disease. Clinical course includes acute and chronic phases, separated by an indefinite period when patients are relatively asymptomatic. The acute phase is usually subclinical with deep parasitemia. In the indeterminate phase, patients have positive serologic and/or parasitological tests but are asymptomatic without radiographic or electrocardiographic manifestations of infection [7]. Among the chronically infected individuals, 25 to 30% develop severe heart disorders [8, 9]. Sixty to 70% of them remain asymptomatic or develop mega syndromes of the esophagus or colon [10]. In these digestive forms, intestinal dilation and muscular hypertrophy of the esophagus or colon are observed in advanced stages of disease, named megaesophagus and megacolon, respectively [11, 12].

The economic impact of Chagas disease is significant and extrapolates the high social cost attributable to chronic patients. Many people at productive age die prematurely, since the available therapeutic drugs only kill the extracellular parasites, and there is not an effective treatment for the disease. It is important to highlight that the damage caused by the parasite is irreversible, leaving consequences that often make it impossible for the patients to perform their daily functions [13–17].

### 1.2. Vector and Parasite

The etiologic agent of Chagas Disease is the flagellate protozoan *Trypanosoma cruzi*, which is mainly transmitted from person to person through blood-sucking insect vectors of the Triatominae subfamily, representing one of the most serious public health concerns in South America [18].

#### 1.2.1. Triatominae Vectors

**Figure 1:**
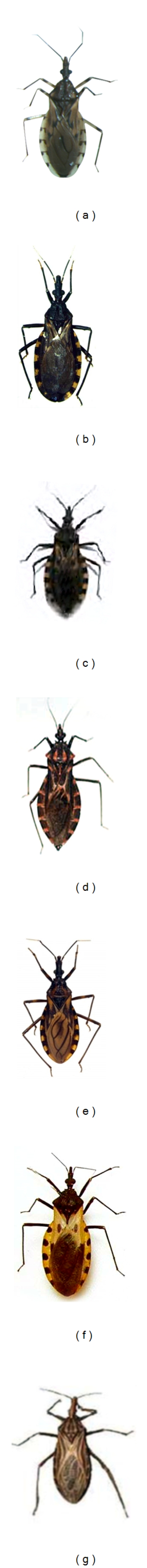
Some of Triatominae insects from the Reduviidae family, which are epidemiologically more significant as potential vectors: (a) *Triatoma sordida*; (b) *Triatoma infestans*; (c) *Triatoma pseudomaculata*; (d) *Panstrongylus megistus*; (e) *Triatoma brasiliensis*; (f) *Triatoma dimidiata*; (g) *Rhodnius prolixus*.

The insect vector of *T. cruzi* is dispersed throughout Latin America and in Brazil is often called “kissing bug” [10]. The potential vectors encompass more than 144 species of Triatominae insects from the Reduviidae family, some of which are epidemiologically more significant such as *Triatoma infestans*, *Triatoma brasiliensis*, *Triatoma dimidiata*, *Rhodnius prolixus*, *Triatoma pseudomaculata*, *Triatoma sordida,* and *Panstrongylus megistus*; Figure 1 [15, 19]. The *Triatoma infestans* is an allochthonous, highly anthropophilic species with the highest rates of infection. It was introduced in São Paulo state from the south of Brazil probably during the 18th century, when there was massive displacement of the agricultural frontier towards the west in search of virgin land for coffee plantation [15].

Currently, the main method of vector control is to spray houses with residual insecticides. However, the occurrence of *T. infestans* populations resistant to pyrethroid compounds in the north of Argentina and Bolivia requires the alternative use of organophosphate insecticides. Unfortunately, these insecticides, although effective, are very toxic and less accepted by the community due to their unpleasant odor [6, 20, 21].

Since *T. infestans* genome has not yet been studied, sequencing of ESTs (expressed sequence tags) is one of the most powerful tools for efficiently identifying large numbers of expressed genes in this insect vector. A total of 826 ESTs were generated, resulting in an increase of 47% in the number of ESTs available for *T. infestans*. These ESTs were assembled in 471 unique sequences, 151 of which represent 136 new genes for the Reduviidae family. Among the putative new genes for this family, an interesting subset of genes involved in development and reproduction, which constitutes potential targets for insecticide development, was identified and described [6].

#### 1.2.2. *Trypanosoma cruzi*

**Figure 2:**
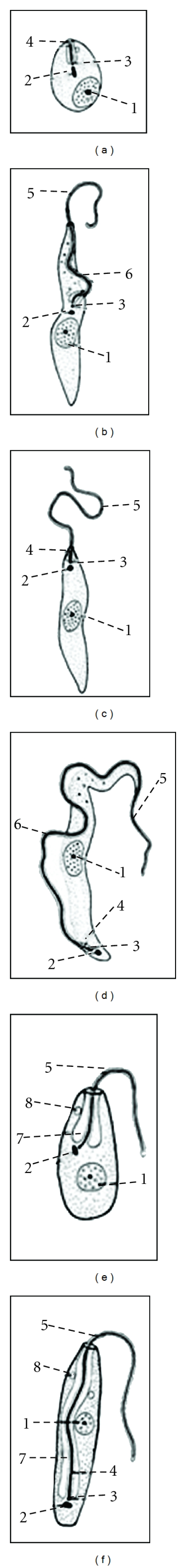
Developmental stages of *Trypanosoma cruzi.* (a) Amastigote, the nonflagellate intracellular morphologic stage; (b) epimastigote, long spindle-shaped hemoflagellate morphologic form equipped with a free flagellum and an undulating membrane that extends one half of the body length. It is found in the vectors responsible for transmitting the *Trypanosoma *species; (c) promastigote, characterized by a free anterior flagellum and the kinetoplast at the anterior end of the body; (d) trypomastigote, leaf-like form with an undulating membrane and often a free flagellum; (e) choanomastigote, barleycorn shaped with a collar-like process where the flagella emerges. Intracellular stage inside the invertebrate host; (f) opisthomastigote, no undulating membrane. Found in the invertebrate host only. (1) Nucleus, (2) kinetoplast, (3) basal body, (4) flagellar pocket (5) flagellum, (6) undulating membrane, (7) mitochondrion, and (8) subpellicular microtubules.

*Trypanosoma cruzi *is a flagellate protozoan of the Kinetoplastida order and Trypanosomatidae family. The parasite's life cycle alternates between vertebrates and insects, with different major principal developmental stages in each host. In the hematophagous vector, the infective replicative epimastigotes (stage with kinetoplast and flagellar pouch in the anterior position of the nucleus), the metacyclic trypomastigotes (kinetoplast in the extremity posterior to the nucleus), and the replicative intracellular amastigotes predominate (rounded form with short inconspicuous flagellum); in the mammalian host the bloodstream trypomastigotes predominate [7] (Figure 2).

Naturally acquired *T. cruzi *infections are initiated in the dermal layers or conjunctival mucosa by infective metacyclic trypomastigote forms that are transmitted by an infected hematophagous triatomine vector [22] and are thereby transformed into amastigotes with the capacity to multiply by simple binary division. Next, they differentiate into trypomastigotes that are released by the host cell into the interstitium and reach the bloodstream and are thus able to invade cells from any tissue to produce a new cycle or be destroyed by host immune mechanisms [7].

## 2. Clonal Histotropic Model of Chagas Disease

There are geographic variations in the prevalence of clinical forms and morbidity of Chagas disease, likely due to both the genetic variation of the *T. cruzi* and the genetic and environmental features of the host [23, 24]. The molecular interaction between the cell surface of the *T. cruzi* clones and the host tissue would be the most likely basis for this tropism. Due to biological polymorphism, different clones in a lineage can present tropism for different tissues, becoming a determinant factor for the disease clinical course due to the clonal repertoire of the infecting lineage and its specific tropisms. This scenario is at the center of what is referred to as the “clonal histotropic model” of Chagas disease [23].

During the Chagas disease acute phase, parasites are present in different organs, but in the chronic phase, they damage specific organs, manifesting genetic heterogeneity among isolates and stocks that may explain the degree of tropism for different organs [33]. The invasion of nonphagocytic host cells by *T. cruzi* depends on parasite surface glycoproteins, and the ability of metacyclic trypomastigotes infectivity varies between different populations of the parasite. These glycoproteins have differential activity in the signaling of Ca^2+^ ions [34].

It has been shown that *T. cruzi* invading mammalian cells binds to the TrkA receptor, the receptor tyrosine kinase widely expressed in the mammalian nervous system, activating TrkA-dependent survival mechanisms, and facilitating its adherence, invasion, and survival [35]. This binding is mediated by the parasite-derived neurotrophic factor (PDNF), a transsialidase located on the surface of the parasite. PDNF in the cytosol of the host cell apparently activates Akt signaling, leading to a suppression of apoptosis [36]. Furthermore, *T. cruzi* transsialidase binds to endothelial cells, triggering activation of NF-kB and leading to protection against apoptosis caused by growth factor deprivation [37].

Murine microarrays studies identified 353 murine genes that were differentially expressed during the early stages of invasion and infection by *T. cruzi* of primary murine cardiomyocytes. Genes associated with the immune response, inflammation, cytoskeleton organization, cell-cell and cell-matrix interactions, apoptosis, cell cycle, and oxidative stress are among those affected during the infection [38]. 

*T. cruzi* is divided into six discrete typing units (DTU): TcI, TcIIa, TcIIb, TcIIc, TcIId, and TcIIe [39–42]. Geographical and epidemiological studies showed that the distribution of TcI and TcII varies geographically. TcI is prevalent in the northern region of Brazil, Central and North America [43, 44], while TcII is found predominantly in the Southern cone countries of Latin America [45]. In Bolivia, the TcIId was found to be the most common [45]. 

The *T. cruzi *kinetoplast minicircle DNA (kDNA) appears to be essential for the study of *T. cruzi *genetic variability from different tissues. PCR (polymerase chain reaction) detection of *T. cruzi *DNA was performed randomly (i.e., without previously determining inflammatory foci) in the esophageal tissue fragments collected from 52 Chagas disease patients. The *T. cruzi* kDNA 330-bp product was detected in 69.2% of esophageal samples, of which 25% were confirmed only after hybridization. The PCR of blood amplified the 330-bp fragment corresponding to *T. cruzi *k-DNA in 90.4% of subjects, of which 83% were detected before hybridization of the amplified products. It was not possible to show a direct relationship between positive tissue and positive blood parasitism because of 40 patients who had tissue parasitism, 92.5% had positive blood PCR, and *T. cruzi *was also detected in the blood of 83.3% of the subjects with negative tissue parasitism. A correlation between the frequency and intensity of the inflammatory process in tissues and the presence of *T. cruzi *could be observed, especially in cases of advanced megaesophagus [46].

Years after, a molecular characterization of parasites evaluated the polymorphisms of the 3_region of the 24S_rRNA gene and the variability of kDNA minicircles of *T*. *cruzi *populations by low-stringency single specific primer LSSP-PCR and data provided a strong correlation between *T*. *cruzi *II and human infection in an endemic in Southeast Brazil area. However, a high degree of variability was observed within *T*. *cruzi *II, as demonstrated by intense kDNA polymorphism among all clinical forms and also within each of them, irrespective of the intensity of pathological processes [47].

*T. cruzi* lineages and (sub)lineages were typified in megacolon samples from 18 Bolivian patients using kDNA probes specific of lineage TcI, TcIIb, TcIId, and TcIIe. The majority of the samples (16/18) were (sub)lineage TcIId positive. However, two samples were positive for (sub)lineage TcIIb. Two synthetic probes discriminated variants of lineage TcIId. Proportion of TcIId variants encountered were 6/16, 6/16, and 4/16, similar to the distribution of Chagasic populations in Bolivia. The data suggest that there is no preferential tropism of one particular lineage or variant of *T. cruzi* II in megacolon pathology [48].

*T. cruzi *kDNA minicircle signatures were evaluated using LSSP-PCR technique in both peripheral blood and esophageal mucosa from Brazilian chronic chagasic patients, with or without megaesophagus, alone or in combination with cardiopathy and megacolon. The study failed to identify a uniform pattern of shared bands between blood and esophageal mucosa samples from individuals with a specific or mixed clinical forms, which suggested occurrence of multiple *T. cruzi *infections with differential tissue tropism. The study evidenced an intense intraspecific variability in the hypervariable regions of the *T. cruzi *kDNA, which has made it impossible to correlate the genetic profile of these hypervariable regions with the Chagas disease clinical manifestations [49].

A study in peripheral blood of 306 Bolivian chronic Chagas disease patients (81 with cardiopathy/150 without cardiopathy; 100 with megacolon/144 without megacolon; 164 with cardiopathy or megacolon/73 indeterminate/17 cases with both cardiopathy and megacolon) successfully amplified the kDNA of *T. cruzi* from 196 samples (64.1%). Of those, 104 (53.3%) were TcIId, 4 (2.0%) were TcI, 7 (3.6%) were TcIIb, 1 (0.5%) was TcIIe, 26 (13.3%) were TcI/IId, 1 (0.5%) was TcI/IIb/IId, 2 (1.0%) were TcIIb/d, and 51 (25.9%) were unidentified. Of the 104 Tc IId samples, three different kDNA hypervariable region patterns were detected, Mn (49.6%), TPK-like (48.9%), and Bug-like (1.5%). However, none of the identified lineages or sublineages was significantly associated with any particular clinical manifestations in the chronic Chagas disease patients [50]. Then, the infection of individual Chagas disease patients may be produced by genetically diverse mixed parasite populations, so it is difficult to establish a relationship between sublineages of parasite and clinical manifestation of Chagas disease.

## 3. Genetic Studies of Chagas Disease Patients

### 3.1. Cardiomyopathies

The pathogenesis of chronic chagasic cardiomyopathy (CCC) is not well understood. Since studies showed that myocarditis is more frequent during advanced stages of the disease and the prognosis of CCC is worse than that of other dilated cardiomyopathies of noninflammatory etiology, it seems that the inflammatory infiltrate plays a major role in myocardial damage [51, 52]. In the last decade, increasing evidence has supported that inflammatory cytokines and chemokines are responsible for the generation of the inflammatory infiltrate and tissue damage. CCC patients have an increased peripheral production of the inflammatory Th1, cytokines IFN-*γ*, and TNF-*α* when compared to patients with the asymptomatic/indeterminate form. Moreover, Th1-T cells are the main producers of IFN-*γ* and TNF-*α* and are frequently found in CCC myocardial inflammatory infiltrate. Furthermore, genetic polymorphisms of cytokine, chemokine, and innate immune response genes have been associated with disease progression [53].

CCC exhibits high levels of circulating procytokines, and lymphocytic infiltrates presenting cytokines (TNF-*α* and IFN-*γ*) are detectable in specimens of cardiac surgical biopsy and tissue [27]. Thus, different polymorphisms in genes of pro- and anti-inflammatory cytokines and others genes involved in host immune response have been evaluated in CCC patients (Table 1). Due to the role of TNF-*α* in the progression of heart failure and to increased levels of plasma and cardiac tissue observed in CCC, Drigo et al. [25] investigated the microsatellite polymorphism (*TNFa2*) and the promoter polymorphism of *TNF-308* (*TNF2*) in 42 patients with severe ventricular dysfunction, according to the presence of the allele *TNF2* promoter or microsatellite *TNFa2-308*. The authors observed that positive patients for the alleles *TNF2* or *TNFa2* exhibited a significantly shorter survival time compared with those carrying other alleles, thus suggesting that the *TNF* genotype can be targeted in therapeutic interventions. However, no association between TNF polymorphism and the severity of the injury was detected [26] when comparing CCC and asymptomatic Chagas disease patients (ASY).

*BAT1* (*HLA-B-associated transcript 1*), a gene with anti-inflammatory activity, and *LTA* (*lymphotoxin alpha*), a pro-inflammatory cytokines member of the TNF family have been associated with coronary artery disease and myocardial infarction [27, 28]. PCR-RFLP (polymerase chain reaction-restriction fragment length polymorphism) studies investigated variants in the promoter region of *BAT1* in positions −22 C/G and −348 C/T [27] and *LTA *in positions −80A3C and −252A3G [28] in CCC and ASY patients. Homozygous *BTA*-22CC constituted 16% of CCC but only 4% of ASY. Similar results were observed for allele −348 C, suggesting that *BAT1* variants, previously associated with reduced expression of *HLA-B-1*, are predictive of CCC evolution. These variants may be less efficient in down-regulation of inflammatory response and may contribute to increased production of pro-inflammatory cytokines in CCC [27]. The homozygous alleles *LTA-80C *and* LTA-252G* were significantly more frequent in CCC than in ASY (47% *versus* 33% and 16% *versus* 8%, resp.). The *LTA* haplotype −80 C–252 G was also associated with susceptibility to CCC. The authors concluded that the study of these genetic variations may help identify Chagas disease patients at increased risk of developing CCC [28].

Because pro-inflammatory cytokines play an important role in CCC, probably SNPs (Single Nucleotide Polymorphisms) in the genes that encode proteins in the TLR (Toll-like receptor) pathway could explain differential susceptibility to CCC among *T. cruzi*-infected individuals. *T. cruzi*-infected individuals who are heterozygous for the MAL/TIRAP S180L variant that leads to a decrease in signal transduction upon ligation of TLR2 or TLR4 to their respective ligand may have a lower risk of developing CCC [29].

Among the cytokines, *TGF*β*1* (transforming growth factor beta multifunctional 1) is essential for the establishment and pathogenesis of *T. cruzi* infection. Several SNPs in this gene able to affect cytokine production have been described, and five of them with functional significance (−988 C/A; −800 G/A; −509 C/T, 10 T/C; 263 C/T) were investigated [30]. The distribution of alleles 10T and 10C showed a significant difference between patients (CCC and ASY) and healthy controls. Additionally, the high frequency of 10 C/C genotype was increased in Chagas disease patients. These data suggest that genetic polymorphisms in codon 10 of *TGF*β**1 may be involved in susceptibility to infection with *T. cruzi* in South American patients [30].

Mannose-binding lectin (MBL) initiates complement on *T. cruzi* through the MBL-associated serine protease 2 (MASP2). Chronic Chagas disease patients were haplotyped [208: including 81 indeterminate and 123 symptomatic (76 with cardiac, 19 with digestive, and 28 with cardio-digestive forms)] for six *MASP2* polymorphisms using PCR with sequence-specific primers and compared with 300 healthy individuals from Southern Brazil. The g.1961795C, p.371D diplotype occurred at a higher frequency among symptomatic patients, compared with the indeterminate group, as well as genotypes with Chagas disease, but not with the g.1945560A in the promoter in cardiac patients. CD haplotypes linked to the p.P126L and p.V377A variants were associated with reduced MASP-2 levels but not reduced MBL/MASP-2/C4 complexes. The authors concluded that MASP2*CD genotypes, most of them generating low MASP-2 levels, are associated with high risk of chagasic cardiomyopathy [31].

Though it is known that the immune system exerts some influence on the resistance against *T. cruzi* infection, its precise role in this process is not well understood. Some *IL-1B* alleles and haplotypes have been associated with susceptibility to inflammatory, autoimmune, and infectious diseases. An investigation of polymorphism (*IL-1B*-511, *IL-1F*10.3 *IL-1RN*.4, *IL-1RN* 6/1, and *IL-1RN* 6/2) was conducted in 86 *T. cruzi* seropositive patients (58 CCC and 28 ASY), 50 seronegative patients with idiopathic dilated cardiomyopathy (IDC) and 109 healthy individuals using RT-PCR allelic discrimination technology. Infected patients presented an increased frequency of the CC genotype of the *IL-1RN*.4 polymorphism when compared to IDC. The C allele or CC genotype of this polymorphism was found increased in CCC when compared with IDC and with controls, suggesting an evident association between the *IL1RN*.4 polymorphism, *T. cruzi* infection, and CCC development [32].

These studies evidence the association of various polymorphisms in genes of immune response and susceptibility to chagasic cardiopathy, so suggesting that host genetic factors may play a role in the underlying mechanisms of disease pathogenesis.

### 3.2. Digestive Tract Alterations

The digestive manifestations of Chagas disease mainly involve megaesophagus and megacolon [3]. The abnormalities of the autonomic enteric nervous system seem to be an essential element in the pathogenesis of chagasic megavisceras. These abnormalities include degeneration and reduction in the number of myenteric plexus that coordinates the motor activity of different segments from the esophagus to the rectum [54]. These lesions occur throughout the digestive tract, but the esophagus and distal portion of the colon are the parts most affected because of the physiology of these segments [55] Furthermore, both regions have a sphincter at their end that must relax by a reflex mechanism.

Van Voorhis and Eisen [56] characterized one cross-reactive antigen (Fl-160), which correspond to an antibody found on the surface of the trypanosome, overlying the flagellum. This antibody cross-reacts with a 48-kD mammalian nervous tissue protein found in sciatic nerve, brain, and myenteric plexi of gut. The myenteric plexi are destroyed by inflammatory infiltrates in Chagas disease, leading to the characteristic megaesophagus and megacolon.

Comparison of the cellular immune response in patients with the digestive and indeterminate forms of Chagas disease on the basis of lymphocyte proliferation and cytokine production after antigen or mitogen stimulation showed no significant differences between patient groups on proliferative response or on TNF-*α* and interleukin (IL)-10 levels, although IL-10 achieves higher levels than TNF-*α* after *T. cruzi* antigen stimulation. IFN-*γ* basal production was significantly higher in the digestive form and IL-4 was significantly higher in patients with megaesophagus when compared with patients with megacolon. These results indicated that patients with the digestive form of Chagas disease do not suffer immune suppression and that the cytokine balance favors a strong inflammatory reaction in patients with the digestive form, which may contribute to lesions of the enteric nervous system [57].

#### 3.2.1. Megaesophagus

Chagasic megaesophagus is consequence of achalasia characterized by the destruction or lack of intramural nerve plexus, which determines the absence of peristalsis and lack of openness of the lower esophageal sphincter in response to swallowing. In consequence, food retention or esophageal stasis occurs, leading to the appearance of chronic esophagitis, acanthosis, paraceratose, and leukoplakia, possibly precancerous lesions [57].

Megaesophagus patients have high variability in esophageal microbiota, which consists primarily of Gram-positive anaerobic bacteria, correlated with the degree of esophageal dilatation [58, 59]. One of the severe late consequences of megaesophagus is the increased risk (3% to 8%) of developing esophageal squamous cell carcinoma (ESCC) [59–61]. Also, ESCC develops in megaesophaus patients at a younger age than in those without this disease [62]. Tumor development is likely related to the prolonged contact of food with the mucosa due to esophageal stasis, increased bacterial growth, and chemical irritation, which results in chronic esophagitis [63].

Idiopathic achalasia and megaesophaus patients, with or without esophageal carcinoma, described changes in expression of proteins such as p53, p16, and MIB (mindbomb homolog) [63–68], chromosomal aneuploidies [59, 69], gene deletions in significant (*TP53)* [69] or marginal levels (*TP63*, *FHIT*, *PIK3CA*, *EGFR*, *CDKN2A*, and *YES*) [70], and gene copy number gain (*PIK3CA*, *TP63*, *FGFR1*, *MYC*, *CDNK2A*, and *NCOA3*) mainly associated with dilation grades III and IV [70]. A strong immunoreactivity of p53 protein was found in patients with idiopathic and megaesophaus, and two of four cases analyzed showed mutations in *TP53* codons 238 and 146 of exons 7 and 5, respectively [63]. The authors suggested that changes in *TP53* in megaesophagus epithelium might be a useful biomarker for identifying individuals with high risk of carcinoma development. In other studies, the frequency of p53 protein immunoreactivity increased significantly when compared to patients with normal esophageal mucosa and achalasia [65, 66]. Thus, these studies suggest that the cell cycle may be altered due to persistent inflammation of mucosa cells, which may arise during dysplasia-carcinoma sequence. 

DNA aneuploidy identified by image cytometry in esophageal specimens of patients with megaesophaus was detected in 27% of 15 patients; similar chromosomal changes also were found in biopsies of megaesophagus, peritumoral tissue, and the center of the tumor of patients with ESCC [59], suggesting that the study of precancerous lesions represents a valuable tool for early diagnosis of esophageal carcinoma. Chromosomal aneuploidies and deletion of *TP53* were also detected in 54% of 40 megaesophaus without ESCC [69]. However, this study has not found mutations in the genes *TP53*, *CDKN2A*, and *FHIT*, which suggested that these events are not common in this lesion [71].

Immunohistochemical studies showed a progressive increase of p53 protein expression in megaesophaus (26.1%) when compared to normal mucosa (7.7%). Also, immunohistochemical stain for p16 and Fhit proteins showed focal and/or diffuse distribution on the basal lamina on the tissue surface for both proteins [68]. However, there is no evidence of alterations in cell kinetics in megaesophaus, since cell proliferation indexes evaluated by Ki67 antigen, and apoptosis by CPP32 antibody was similar to normal mucosa [72]. These studies have shown that p53 overexpression is involved in the initial steps of esophageal carcinogenesis, supporting further evaluation of this marker in precursor lesions [68].

Despite the scarceness of genetic studies in megaesophaus, the available data supports occurrence of genetic changes associated with regulation of the cell cycle control, similarly to esophageal carcinoma, thus indicating that these alterations can be involved in the progression of esophageal carcinogenesis from precursor lesions.

#### 3.2.2. Megacolon

Chagasic megacolon is the large intestine dilation and elongation, mainly due to changes in the viscera intrinsic innervation, with consequent morphological and functional disorders [73]. The megacolon, a complication of Chagas disease is relatively common and has been considered the most common surgical disease of the colon [73]. It is difficult to detect natural megacolon, perhaps because of its slower growth rate, milder symptoms, and tendency to manifest later in life, or perhaps due to the fact that the patient supports better symptoms of constipation than dysphagia. However, it is estimated that 10 to 12% of Chagas disease cases (around 30,000 per year) develop megacolon [3].

Megacolon is slightly predominant in men, between 20 and 60 years of age, and peaking around 40–50 years. The disease is mostly acquired in rural areas due to contact of individuals with triatomid feces. The disease is under global control, and the current frame shows a prevalence of 0.13% in endemic areas, data obtained by serological survey [73].

da Silveira et al. [74] hypothesized that enteric glial cells may be involved in the modulation of enteric inflammatory responses or even control the colon's dilation. Neuronal loss is similar in dilated and nondilated portions of megacolon; moreover, neuronal destruction present in megacolon is preceded by glial component loss. The nondilated portion of megacolon exhibited increased expression of glial fibrillary acidic protein comparable with the dilated portion and also to the noninfected patients. These results suggest that glial fibrillary acidic protein enteric glial cells prevent dilatation of the organ and protect the enteric nervous system against the inflammatory process and neuronal destruction, preventing the destruction from expanding to unaffected areas of the colon [75]. Subjects with megacolon had significantly more CD-57 natural killer cells and TIA-1 cytotoxic lymphocytes within enteric ganglia, but numbers of CD-3 and CD-20 immunoreactive cells were not significantly elevated. The innervation of the muscle was substantially reduced to about 20% in megacolon, but asymptomatic seropositive subjects were not different of seronegative controls. Glial cell loss occurred equally in symptomatic and unaffected seropositive subjects, although the proportion with glial fibrillary acidic protein was greater in seropositive, nonsymptomatic subjects [74].

Other molecular markers have been described in megacolon. For example, dilated portions of colon present with high levels of substance P, a neurotransmitter involved in pain transmission that causes rapid contractions of the gastrointestinal smooth muscle and modulates inflammatory and immune responses, and low levels of the NK1 receptor. Conversely, nondilated colon and noninfected individuals present low levels of substance P and high levels of NK1 receptor, which may indicate a neuroimmune relationship occurring in Chagas disease [76].

It is believed that the presence of positive Foxp3 (a protein involved in immune system responses) cells [Foxp3(+)] may help control the inflammatory process through the management of lymphocyte migration. Chagas disease patients without megacolon presented with an increased concentration of Foxp3(+) cells in all colon layers compared with megacolon patients and noninfected individuals. These cells were situated mainly near the blood vessels and rarely were associated with the inflammatory foci; consequently, they seemed to prevent neuronal destruction and megacolon development [77].

The expression of molecules responsible for activation of T cells by neurons and enteric glial cells was investigated and shows only enteric glial cells of Chagasic patients with megacolon expressed HLA-DR complex class II and costimulatory molecules [78]. Therefore, the development of megacolon after acute infection with *T. cruzi* is associated with a maintained invasion of enteric ganglia with cytotoxic T cells and loss of muscle innervation. However, changes in glial cell numbers are not associated with progression of enteric neuropathy.

## 4. The Brazilian Survey on Chagas Disease

The main results of three large national surveys on Chagas disease (entomologic, seroprevalence and electrocardiographic) carried out in Brazil from late 1970s to the early 1980s served as baseline for the definition of the control measures adopted in the country [14]. The proportion of infected people was much higher in areas where *Triatoma infestans*, the most efficient vector of Chagas disease among the five principal species involved in transmission at that time, was predominant. Similar result was observed in places where *Triatoma sordida* was dispersed, mainly in the country's central region, which corresponds to its native area. These findings are likely related to the colocalization of the geographic distribution of both vectors, since *T. sordida* is not considered an important player in Chagas disease transmission. In the semiarid, endemic, Brazilian Northeastern area, rates of human infection by *Triatoma brasiliensis* and *Triatoma pseudomaculata* were much lower, although both vectors may have some relevance in the maintenance of the disease. As for areas with *Panstrongylus megistus*, human infection varied according to the levels of vector domiciliation. When *Panstrongylus megistus* is resident this can demonstrate that it has an important role in domestic transmission of *T. cruzi*, as in the humid coast of the northeast. In some parts of the Bahia state, *Panstrongylus megistus* represented the exclusive vector of the disease. Based upon the results of the seroprevalence survey, an electrocardiographic study was carried out in 11 Brazilian states, which showed marked differences in the presence of cardiac alterations among different geographical areas of the country [14].

A survey for seroprevalence of Chagas disease was held from 2001 to 2008 in a representative sample of Brazilian children (up to 5 years old) living in rural areas of all Brazilian states but Rio de Janeiro. Blood on filter paper was collected from 104,954 children and screened in a single laboratory with two serological tests: indirect immunofluorescence and enzyme-linked immunoassay. All samples with positive or undetermined results, as well as 10% of all negative samples, were submitted to a quality control reference laboratory, which performed both tests a second time, in addition to the western blot assay of TESA (trypomastigote excreted secreted antigen). All children with confirmed positive result (*n* = 104, prevalence = 0.1%) had a followup visit and were submitted to a second blood collection, this time a whole blood sample. In addition, blood samples from the children's mothers and relatives were collected. The infection was confirmed in only 32 (0.03%) of those children. From those, 20 (0.025%) had maternal positive results, suggesting congenital transmission; 11 (0.01%) had noninfected mothers, indicating a possible vectorial transmission; and in a single child, whose mother had died, the transmission mechanism could not be elucidated. In further 41 visited children, the infection was confirmed only in their mothers suggesting passive transference of maternal antibodies; in other 18, both child and mother were negative; and in 13 cases, the subjects were not localized. The 11 children that acquired the infection presumably through the vector were distributed mainly in the northeast region of Brazil (states of Piauí, Ceará, Rio Grande do Norte, Paraíba and Alagoas), in addition to one case in Amazonas (north region) and another in Parana (south region) [79].

Remarkably, 60% of the 20 probably congenital transmission cases were from a single state, Rio Grande do Sul, with the remaining cases distributed in numerous other states. This is the first report demonstrating regional geographical differences in the vertical transmission of Chagas disease in Brazil, and the hot spot in Rio Grande do Sul probably reflects the predominant *T. cruzi* group IId and IIe (now TcV and TcVI) found in this state. Overall, these results show that the regular and systematic control programs against the transmission of Chagas disease, together with socioeconomic changes observed in Brazil in the last decades, were effective in interrupting the vectorial transmission of Chagas disease in the country. Furthermore, these data reinforce the need for maintenance of the control programs in order to consolidate this major advance in public health [79, 80].

## 5. Conclusions

Chagas disease is still active in various countries of Latin America and affects a great number of individuals who undergo undiagnosed until they manifest the typical advanced symptoms or are affected by other concomitant pathologies. There has been significant progress in understanding the biological and genetic diversity of the parasite, as well as the population polymorphisms associated with susceptibility to this disease. However, many other aspects such as host-parasite interactions, genetic mechanisms of cellular interaction, genetic variability, and tropism are not enough known. Further investigations on these aspects are necessary to clarify the *T. cruzi's* mechanisms of action and support robust efforts on public health to eradicate the disease.

## Figures and Tables

**Table 1:** Genetic polymorphisms in Chagas disease cardiomyopathy.

Genes	SNP	Effect	Association to cardiomyopathy		References
			Patients	Results	
*TNF*α**		Pro-inflammatory cytokines			[25, 26]
	−308 G/A (*TNF2*) *TNFa2 *	High *TNF-a* production	42 CCC	Allele *TNF2* or microsatellite *TNFa2 *associated with worse prognosis Shorter survival time compared with those carrying other alleles	[25]
			166 CCC	No significant differences between CCC and ASY patients	[26]
			80 ASY		
				Lack of association of *TNF* polymorphisms with CCC development or to progression cardiomyopthy	

*BAT1*	−22 C/G −348C/T	Anti-inflammatory activity associated with reduced expression of *HLA-B-1 *	154 CCC 76 ASY	Homozygous −22 CC and −348 CC more frequent in CCC than in ASY Both variants are predictive of CCC evolution	[27]

*LTA*	+80 A/C +252 A/G	Pro-inflammatory cytokines	169 CCC 76 ASY	Homozygous +*80* *CC *and* +252* *GG* more frequent in CCC than in ASY Haplotype +80 C +252 G associated to CCC susceptibility	[28]

*TLR*	*TLR1* *TLR2* *TLR4* *TLR5* *TLR9*	Pathogen recognition receptors Component of innate immunity	169 CCC 76 ASY	*TLR *polymorphisms did not show major risk factors for the development of CCC	[29]

*MAL/TIRAP*		Encodes an adaptor protein for TLR	169 CCC 76 ASY	Heterozygous *MAL/TIRAP *S180L associated with lower risk of developing CCC	[29]

*TGF*β*1*	−988 C/A −800 G/A −509 C/T 10 T/C 263 C/T	Multifunctional cytokine	172 CCC 175 ASY 279 health control patients	−988 C/A and 263 C/T were not detected −800 A was uncommon, and −509 C/T was not associated with Chagas disease Allele C and the genotype C/C at codon 10 were associated with Chagas disease patients Allele C may be a risk factor for genetic susceptibility to Chagas disease patients	[30]

*MASP2*	Six SNP	Involved in the complement system	208 Chagas disease 300 health control patients	MASP2 *CD genotypes were associated risk of CCC	[31]

*IL-1B*	*IL-1B-511* *IL-1F10.3* * IL-1RN.4* * IL-1RN 6/1* *IL-1RN 6/2*	Pro-inflammatory cytokines Receptor antagonist	58 CCC 28 ASY 50 IDC	C allele or CC genotype of the *IL-1RN.4* was increased in CCC when compared with IDC and health control patients, evidencing association between this polymorphisms and CCC development	[32]

ASY: asymptomatic patients; CCC: chronic Chagasic cardiomyopathy patients; IDC: idiopathic dilated cardiomyopathy patients.
